# Genetic Basis of Phenotypic Differences Between Chinese Yunling Black Goats and Nubian Goats Revealed by Allele-Specific Expression in Their F1 Hybrids

**DOI:** 10.3389/fgene.2019.00145

**Published:** 2019-03-05

**Authors:** Yanhong Cao, Han Xu, Ran Li, Shan Gao, Ningbo Chen, Jun Luo, Yu Jiang

**Affiliations:** ^1^Key Laboratory of Animal Genetics, Breeding and Reproduction of Shaanxi Province, College of Animal Science and Technology, Northwest A&F University, Yangling, China; ^2^Guangxi Key Laboratory of Livestock Genetic Improvement, The Animal Husbandry Research Institute of Guangxi Zhuang Autonomous Region, Nanning, China

**Keywords:** allele-specific expression (ASE), Chinese Yunling black goat, Nubian goat, whole genome sequencing, RNA-seq

## Abstract

Chinese Yunling black goats and African Nubian goats are divergent breeds showing significant differences in body size, milk production, and environmental adaptation. However, the genetic mechanisms underlying these phenotypic differences remain to be elucidated. In this report, we provide a detailed portrait of allele-specific expression (ASE) from 54 RNA-Seq analyses across six tissues from nine F1 hybrid offspring generated by crossing the two breeds combined with 13 genomes of the two breeds. We identified a total of 524 genes with ASE, which are involved in bone development, muscle cell differentiation, and the regulation of lipid metabolic processes. We further found that 38 genes with ASE were also under directional selection by comparing 13 genomes of the two breeds; these 38 genes play important roles in metabolism, immune responses, and the adaptation to hot and humid environments. In conclusion, our study shows that the exploration of genes with ASE in F1 hybrids provides an efficient way to understand the genetic basis underlying the phenotypic differences of two diverse goat breeds.

## Introduction

A domestic species can present diverse phenotypic differences due to the adaptation to local environments and artificial selection. Yet, it has been difficult to identify the causative genes that contribute to these phenotypic differences. Some studies have relied on genomic selection signals (Dong et al., 2012; Benjelloun et al., 2015). However, the identified selection signals generally contain a high proportion of background noise. Comparative transcriptome analysis of breeds with distinct traits is another frequently used approach (Hayano-Kanashiro et al., 2009; von Heckel et al., 2016). However, the resulting differentially expressed genes reflect both cis-acting and trans-acting regulatory variations, thus presenting little power to characterize the genetic architecture and identify causative genes. With the development of sequencing-based methods to study the transcriptome, it is possible to make use of natural sequence variation to trace and quantify allele-specific expression (ASE) in F1 hybrid individuals generated from crosses of two different lines of interest (Crowley et al., 2015; Aguilar-Rangel et al., 2017). Characterization of ASE in F1 material avoids the problem of comparing parents that may differ dramatically in their growth and development by evaluating both alleles within the same cellular environment, directly revealing cis-acting genetic variation related to transcript accumulation.

The black goats in Southwest China are characterized by a tolerance to crude feed, a higher resistance to parasitic diseases, and thinner muscle fibers (Miao et al., 2015). However, the growth rate of these black goats is much slower than that of commercialized breeds (Zhao et al., 2011) improved by European countries. Nubian goats, a popular commercialized breed, exhibit high feed efficiency and a fast growth rate but are susceptible to parasites (Kholif et al., 2017; Rahmatalla et al., 2017), which are common in the hot and humid environment of South China. In the past decades, Nubian goats have been continuously imported into China to improve the production performance of local breeds (Yuan et al., 2017). Understanding the genetic basis underlying the distinct phenotypes of these two breeds will be a perquisite for new breed selection and customizing strategies for cross breeding.

To understand the genetic mechanisms underlying the phenotypic differences between these two breeds, an F1 hybrid population was generated by crossing female Chinese Yunling black goats and male Nubian goats, and the transcriptomes of the F1 hybrids were analyzed in six tissues (liver, bone, muscle, fat, skin, and mammary gland tissues) to detect ASE. Combined with the selection signals identified in Chinese Yunling black goats and Nubian goats, we provide further insights into the genomic contributions underlying the phenotypic diversity between these two goat breeds.

## Materials and Methods

### Sample Collection

Six Chinese Yunling black ewes and four Nubian rams were selected to produce nine F1 hybrids. Nine female F1 hybrids (three from each cross) were slaughtered after being stunned by high voltage electricity. Liver, bone, muscle, fat, skin, and mammary gland tissues from all nine hybrids were rapidly dissected, snap frozen in liquid nitrogen, and stored at −80°C until use. In addition, three of the individuals additionally collected horns, hooves, and rumen for the calculation of reads counts and genotype judgment. For each tissue sample, two replicates were collected simultaneously. Blood samples were collected from the parents (six Chinese Yunling black ewes and four Nubian rams) and three female F1 hybrids.

### DNA Extraction and DNA Sequencing

Genomic DNA was extracted from blood samples using a Tiangen DNA isolation kit (Tiangen Biotech, Beijing, China). At least 6 μg of genomic DNA from each sample was used to construct a sequencing library following Illumina instructions. Paired-end sequencing libraries with an insert size of approximately 500 bp were sequenced using an Illumina HiSeq 2000 (Berry Genomics Company).

### RNA Extraction and RNA-Sequencing

Total RNA was extracted using TRIzol (Invitrogen, Carlsbad, CA, USA) following the manufacturer protocols. RNA quality was measured using an Agilent 2100 Bioanalyzer. All samples had an RNA integrity Number (RIN) ≥7. Library construction and sequencing were performed according to Illumina instructions. mRNA was isolated from DNA-free total RNA using the Dynabeads mRNA DIRECT Kit (Invitrogen) and fragmented. First-strand cDNA was generated using Random Primer p(dN)6 and Superscript III, after which second-strand cDNA synthesis and adaptor ligation were performed. cDNA fragments of 400–500 bp were isolated. The library was sequenced using the Illumina X-ten platform to generate 150 bp paired-end reads (Berry Genomics Company).

### Genomic Sequence Analysis

Before alignment, the raw data were processed to filter out adaptors and low-quality reads. High-quality clean reads from the DNA sequencing of parents were aligned to the goat reference genome (Bickhart et al., 2017) using BWA software (Li and Durbin, 2009). We then assigned SNPs to the two groups using the Genome Analysis Toolkit (GATK, v3.2-2) (McKenna et al., 2010) to discriminate the parents from both lines. Next, we filtered low-quality sites using the parameter QUAL < 30. All the assigned variants were annotated using the package ANNOVAR (Version: 2013-08-23) (Wang et al., 2010).

### Analysis of Selective Sweeps

We performed a selective sweep analysis by calculating the genetic differentiation (Fst) and heterozygosity (Hp) of each 150 KB genome window and 75 KB step length. Fst was calculated using VCFtools (Kofler et al., 2011), and Hp was calculated as described previously (Rubin et al., 2012). The Hp and Fst values were converted to a standard normal distribution, denoted by ZHp and ZFst. In addition, regions that exhibited low Hp and high Fst values were screened as candidates. To understand the biological functions of genes within candidate regions, GO analysis was performed.

### Transcriptome Mapping and Quantification of Expression

Clean reads were mapped to the CHIR_3.0 reference genome (Bickhart et al., 2017) using STAR with default options. Next, the unmapped reads were remapped to the genome using Hisat2 (v 2.0.3) (Pertea et al., 2016). The assignment of reads to genes was performed using StringTie (Pertea et al., 2016). The expression levels of the protein-coding genes were quantified using the R package “Ballgown” (Pertea et al., 2016).

### ASE Analysis

Allele counts were retrieved using a homemade Python scripts (Supplementary File 1: GetSnpCountFromBam.py) which calculates allele counts at SNP positions. Heterozygous sites with individual allele read depth <20 and total (both alleles) read depth <50 were filtered out. A binomial test and Benjamini-Hochberg FDR correction were performed. Cut-off criteria of allele ratio >0.7 or <0.3 and FDR <0.05 were used to identify significant allelic imbalances. Previously identified imprinted genes obtained from an online database (http://www.geneimprint.com/site/home) were excluded from our final gene set.

### Gene Ontology Analysis

GO pathway enrichment analyses were performed to identify enriched functions in KOBAS3.0 (http://kobas.cbi.pku.edu.cn/index.php). We converted the goat gene symbol IDs into human homologous gene symbol IDs using Blastp before performing GO pathway analyses, as the goat gene annotations in the KOBAS3.0 database were inadequate. We set the EASE value to 0.05 for the enrichment analysis.

## Results

### Genomic Variants of Chinese Yunling Black Goats and Nubian Goats

**Figure 1:**
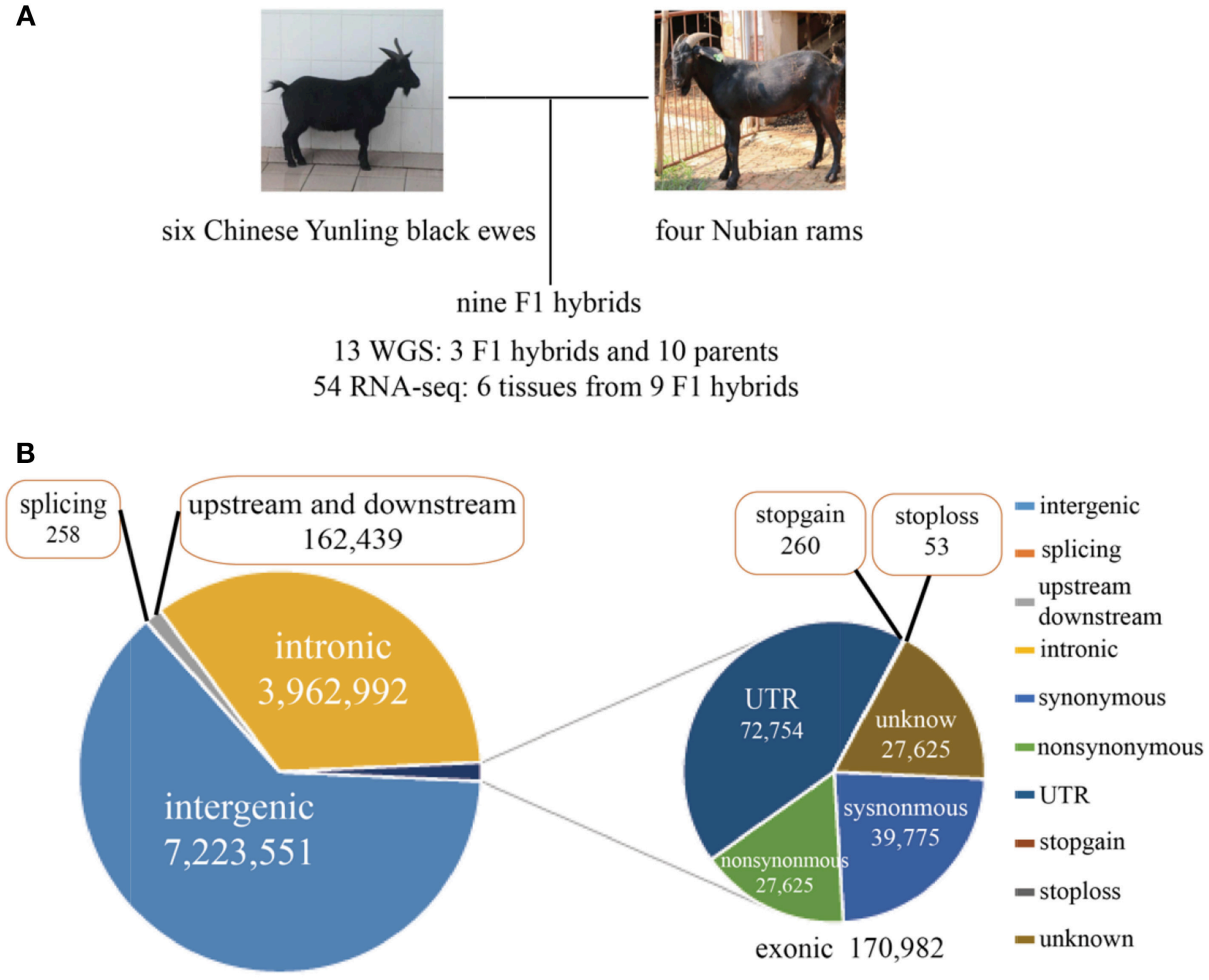
**(A)** Experimental design and brief data summary. **(B)** Statistics regarding the location and mutation type of the whole-genome SNPs.

Six Chinese Yunling black goats, four Nubian goats and three F1 hybrids were selected for genome resequencing (Figure 1A). The genome resequencing achieved an average depth of 15X and a mapping rate of 99.54%. A total of 11.52 million SNPs were found to differ between the parents of each breed, and 309,984 SNPs were expressed. Among the total discriminating SNPs, 0.85% were detected in coding regions (Figure 1B; Supplementary File 2). In addition, we detected 313 SNPs in termination codons and 258 SNPs in splice sites. We found that 24,701 genes were annotated genes with at least one discriminating SNP.

Interestingly, we found 7,365 genes that contained more than 100 SNPs, implying that these genes were highly diverse and might be particularly susceptible to artificial selection (Figure 1, Supplementary Figure 1A). The proportion of substitution transitions (69.4%) was much higher than that of transversions (30.7%) (Supplementary Figure 1B). The transition:transversion ratio was 2.26:1, which is similar to that found in other goat studies (Guan et al., 2016).

### Genome-Wide Selective Sweep Analysis

**Figure 2:**
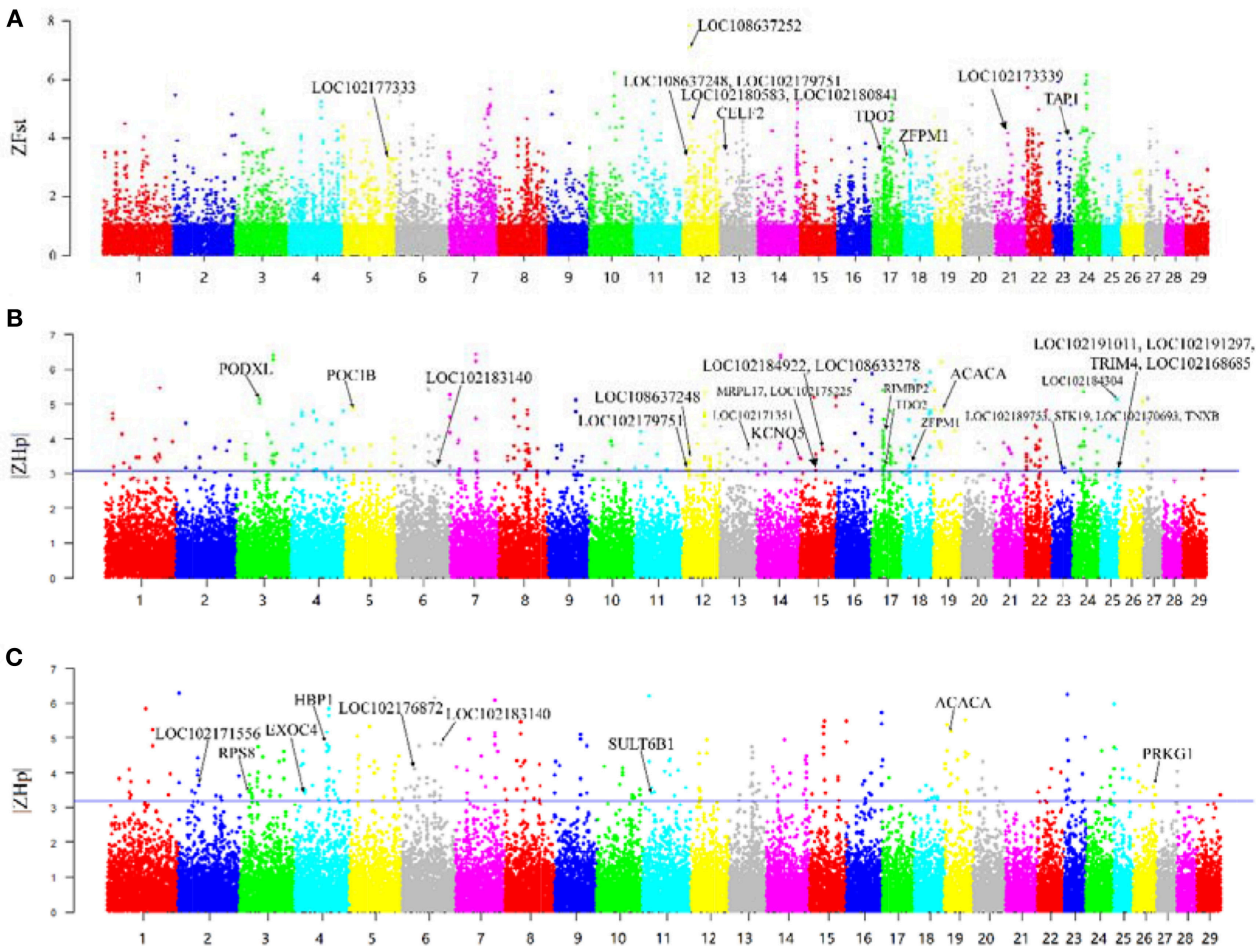
Overview of selective sweeps in the Nubian and Yunling black goat breeds based on ZFst and ZHp values. The labeled genes in bold characters represent those genes with selective signals that overlap with ASE genes. **(A)** ZFst values between Nubian and Yunling black goat breeds. Bold names are the ASE genes contained in the maximum Z-Fst in the 100 kb window. **(B)** ZHp value of Nubian goats. Bold names are the ASE genes contained in the minimum |ZHp| group of Nubian goats in the 100 kb window. **(C)** ZHp value of Yunling Black goats. Bold names are the ASE genes contained in the minimum |ZHp| group of Yunling Black goats in the 100 kb window.

F-statistic (Fst) scores were calculated to measure the signature of selection between the Yunling black goats and Nubian goats. We scanned the autosomes with a nonoverlapping 100 kb window and calculated the Fst value for each window. We focused on the regions with extremely high Z-transformed Fst values (Top %1) in the genome-wide empirical distribution. In total, 250 putative selective sweep regions containing 521 candidate genes were identified (Figure 2A; Supplementary File 3).

The region with the strongest differentiation signal [ZFst = 7.85] between the two breeds was the 13.73–13.95 MB region of chromosome 12, which contained *LOC108637252*/*LOC108637248*/*LOC102180841*/*LOC102180583* (*MRP4*). The product of this region protects cells against toxicity by acting as an ion efflux pump, in addition to influencing dendritic cell migration (Li et al., 2017). We also identified several genes showing differentiation, including *CELF2, TDO2, ZFPM1, TAP1, LOC102177333* (*CYP2D6*), and *LOC102173339* (*CYP8B1*).

Heterozygosity (Hp) was also used to detect putative selective sweeps. The distribution of the observed Hp values and the Z transformations of Hp and ZHp are plotted in Figure 2. We searched the regions with the lowest heterozygosity (top 1% based on |ZHp| scores), which yielded a total of 275 putative selective sweep regions containing 785 candidate genes in Nubian goats (Figure 2B) and 270 putative selective sweep regions containing 854 candidate genes in Chinese Yunling black goats (Figure 2C and Supplementary File 3).

We observed high ZHp values (ZHp = 6.40) across the *PCDHB* (protocadherin B) gene family (Figure 2B) in Nubian goats and for the *UBR4* and *EMC1* genes (ZHp = 6.28) in Chinese Yunling black goats (Figure 2C).

### Transcriptome Characterization of F1 Hybrids

To detect genes with ASE and infer the existence of cis-regulatory variants, we combined the RNA-Seq data from six tissues (liver, bone, muscle, fat, skin, and mammary gland tissues) and the whole-genome sequencing results from three female F1 hybrids (Figure 1A and Supplementary File 2). The whole-genome resequencing data were used to exclude possible base changes in RNA sequences resulting from RNA editing.

The greatest number of ASE SNPs (2,685) were detected in the mammary gland, while 1,556 were detected in muscle Table 1. Most of the ASE SNPs were located in annotated genes (liver 79.6%, bone 81.7%, muscle 87.5%, fat 77.8%, mammary gland 80.9%, and skin 81.4%) (Table 1). The percentage of synonymous regions was over 50% in the six tissues, and the ratio in muscle was highest, reaching 64%(Table 2).

**Table 1:** Annotation of SNPs with ASE from six tissues.

**ASE SNP annotation**	**Liver**	**Bone**	**Muscle**	**Fat**	**Mammary**	**Skin**
Total	2,341	2,004	1,556	2,349	2,685	2,362
SNPs in annotated genes	1,864	1,637	1,361	1,827	2,173	1,923
Exon	885	1,073	1,103	1,347	1,353	1,493
Intron	978	562	257	477	817	429
Splice region	1	2	1	3	3	1
Intergenic	381	257	146	406	400	325
Upstream and downstream	96	110	49	116	112	114

**Table 2:** Mutation statistics of SNPs with ASE located on exons.

	**Liver**	**Bone**	**Muscle**	**Fat**	**Mammary**	**Skin**
Synonymous	230	291	323	362	384	397
Non-synonymous	189	266	182	244	277	244
Stopgain	0	0	0	0	0	0
Stoploss	2	0	0	0	0	0

The genes we identified that had ASE may have included imprinted genes. We therefore collected the imprinted genes of human, mouse, cattle and sheep (Supplementary Table 1) from a publicly available database (http://www.geneimprint.com/site/home) and excluded these from our results. In this way, we finally identified 524 genes with ASE in the six tissues, ranging from 78 in muscle to 144 in liver (Table 3). The greatest number of ASE genes comprised protein-coding genes, whose proportion in the six tissues of all hybrids was above 90%, followed by noncoding RNAs (Table 3). Using an FPKM > 0.01 as a threshold, the average expression level of the ASE gene was significantly higher than the average expression of the normal gene in the same tissue (Supplementary Table 2).

**Table 3:** Encoding type for genes with ASE.

	**Protein coding**	**Noncoding RNA**	**Pseudogene**	**Total**
Liver	135	8	1	144
Bone	101	3	3	107
Muscle	74	4	0	78
Fat	92	2	3	97
Mammary	114	7	3	124
Skin	121	4	2	127

### Functional Annotation of ASE Genes

To explore the tissue specificity of the genes with ASE, functional enrichment analyses were performed. In bone, 276 Gene Ontology (GO) terms were significantly enriched in 77 ASE genes (*P* < 0.05), most of which were associated with hematopoietic or lymphoid organ development (Table 4 and Supplementary File 3). There were 332GO terms enriched in 72 ASE genes in muscle (*P* < 0.05) (Supplementary File 4), which were mainly involved in striated muscle cell development, the actin cytoskeleton, muscle cell differentiation, actin-mediated cell contraction, muscle fiber development, striated muscle thin contraction, and muscle tissue development (Table 4). The results revealed that 385 GO terms were significantly enriched in 82 ASE genes in fat tissue (*P* < 0.05) (Supplementary File 4). Interestingly, lipid-related processes (lipid localization, lipid binding, and lipid metabolic processes) were significantly enriched in 11 ASE genes, including ABCA6, LBP, SEC14L2, SERINC2, ACACA, CD36, AADAC, C3, LGALS12, PLA2G16, and MFGE8 (Table 4). The liver is an important metabolic organ, for which 10 metabolic-related GO terms were found, including the metabolic processes of small molecules, organic acids, cellular functions, carboxylic acid, oxoacid, monocarboxylic acid, cofactors, organonitrogen compounds, cellular lipids, and coenzyme function (Table 4; Supplementary File 4). There was only one significantly enriched GO term for bone and skin, respectively, and none was significantly enriched for mammary gland.

**Table 4:** GO analysis of the genes with ASE in difference tissues.

**Tissue**	**GO Term**	**Gene number**	**Corrected *P*-value**
Bone	Hematopoietic or lymphoid organ development	8	1.88E-03
	Hemopoiesis	7	5.91E-03
Muscle	Striated muscle cell development	10	1.77E-11
	Actin cytoskeleton	13	4.02E-10
	Muscle cell differentiation	11	5.19E-09
	Actin-mediated cell contraction	6	1.88E-06
	Muscle fiber development	5	3.37E-06
	Striated muscle contraction	6	1.68E-05
	Muscle tissue development	6	7.06E-04
Fat	Lipid localization	5	6.23E-03
	Lipid binding	6	1.30E-02
	Lipid metabolic process	8	2.84E-02
Skin	Keratin filament	11	2.32E-13
Liver	Small molecule metabolic process	43	1.39E-23
	Organic acid metabolic process	31	4.47E-21
	Cellular metabolic process	78	1.18E-18
	Carboxylic acid metabolic process	26	7.63E-17
	Oxoacid metabolic process	26	8.50E-17
	Monocarboxylic acid metabolic process	21	2.16E-15
	Cofactor metabolic process	17	1.63E-13
	Organonitrogen compound metabolic process	33	2.65E-13
	Cellular lipid metabolic process	22	1.74E-11
	Coenzyme metabolic process	14	3.52E-11

Among the six tissues, the ASE genes of the liver presented the highest tissue specificity (77.8%), followed by those in the skin (71.7%), bone (68.2%), muscle (67.9%), and mammary tissue (52.4%). In contrast, fat exhibited a lower level of tissue specificity (45.4%). Supplementary Table 3 shows the status of the overlap of ASE genes in different tissues. For example, *HLA-A, HLA-B, HLA-DQA1, HLA-DQB1*, and *LOC106503915* were detected in all tissues Supplementary Table 3. The *HLA* genes encoded major histocompatibility complex (*MHC*) class I proteins in the context of specific cell surfaces (Valenzuela-Ponce et al., 2018), involving *HLA-A, HLA-B, HLADRB1, HLA-DQA1*, and *HLA-DQB1* (Emerson et al., 2017). *HLA* played a major role in the control of the immune response and its associations with a wide variety of immunological and infectious disorders, such as type I diabetes, multiple sclerosis, rheumatoid arthritis, Grave's disease, ankylosing spondylitis, and systemic lupus erythematosus (Spínola et al., 2016).

### Potential Effects of ASE Genes Under Selection

We also observed several ASE genes under selection. The range of selected ASE genes in six tissues was 5 to 13. Among these genes, we highlight the Ribosomal protein S8 (*RPS8*) gene. *RPS8* was detected in four tissues: skin, bone, mammary tissues and muscle tissue. It also showed the lowest level of heterozygosity in the selected region in Yunling black goats (|ZHp| = 3.46). *RPS8* has been used to develop a species-specific PCR-RFLP diagnostic tool for ovine babesiosis and theileriosis, which are hemoprotozoal diseases that cause economic losses among sheep and goats in tropical and subtropical regions (Tian et al., 2013).

Another ASE gene, Multidrug resistance protein 4 (*MRP4*), was specifically expressed in the bone, which contained 32 SNPs (29 exonic and three intronic SNPs). Based on the results for *MRP4*, Nubian goats with the highest Fst value [Z(Fst) = 7.08] and Hp value (|ZHp| = 3.33) were selected. *MRP4* has been identified as an important transporter for signaling molecules, including cyclic nucleotides and several lipid mediators in platelets. *MRP4* is known to play a critical role in the elimination of numerous drugs, carcinogens, toxicants, and their conjugated metabolites and is expressed at the basolateral surface of hepatocytes, which can facilitate cellular efflux to sinusoidal blood for entry into the systemic circulation (Li et al., 2017).

**Figure 3:**
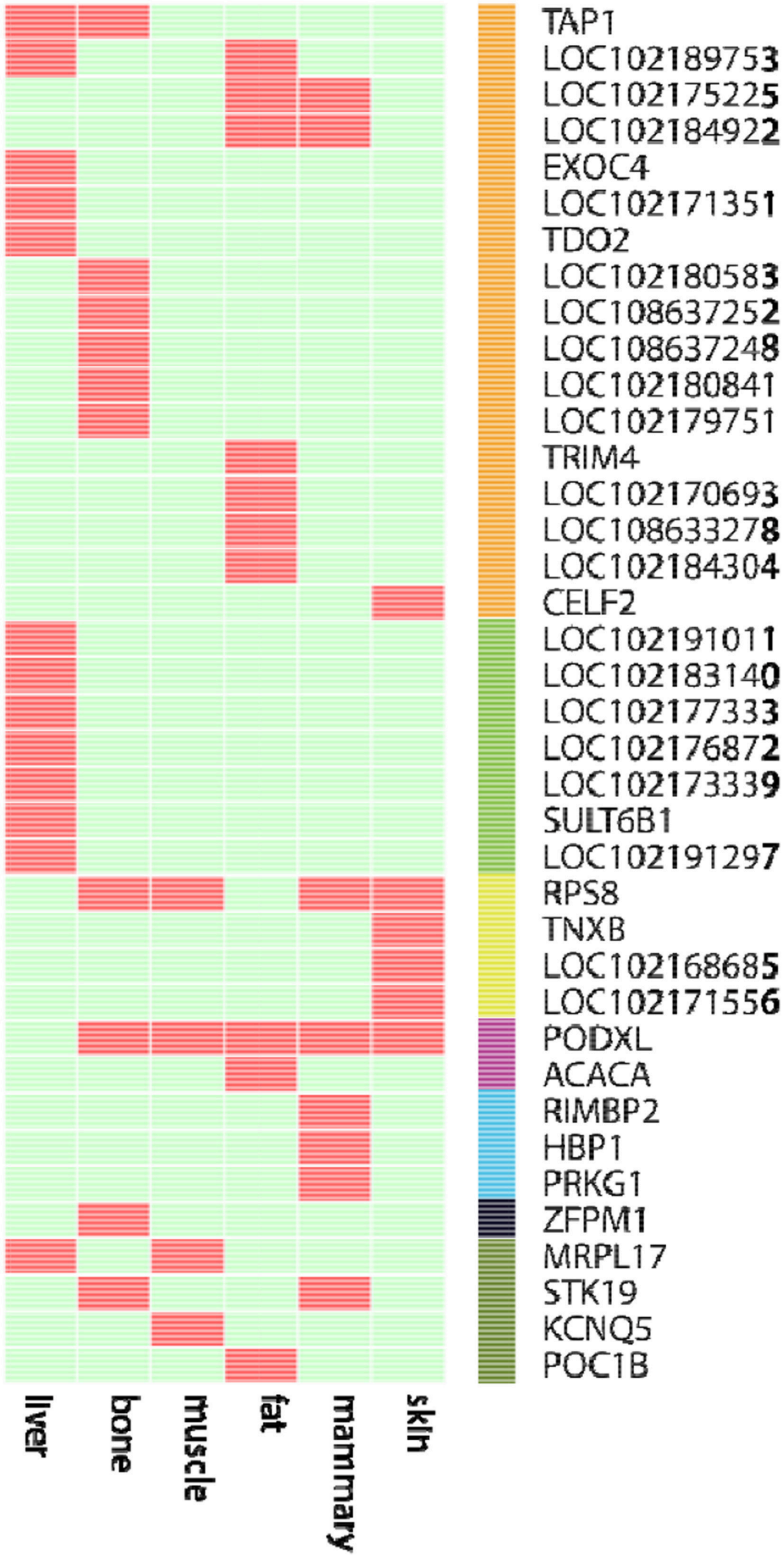
The 38 core-ASE genes and their functions. Red and light green indicate the presence and absence, respectively, of genes with ASE (rows) in the corresponding tissues (columns). The right bar indicates the biological functions of the corresponding genes (Orange: immune responses; green: metabolism; yellow: adaptation to hot and humid environments; purple: functions associated with body measurements and weight; cyan: cell regulation metabolism; black: hematopoiesis; dark green: undefined).

**Figure 4:**
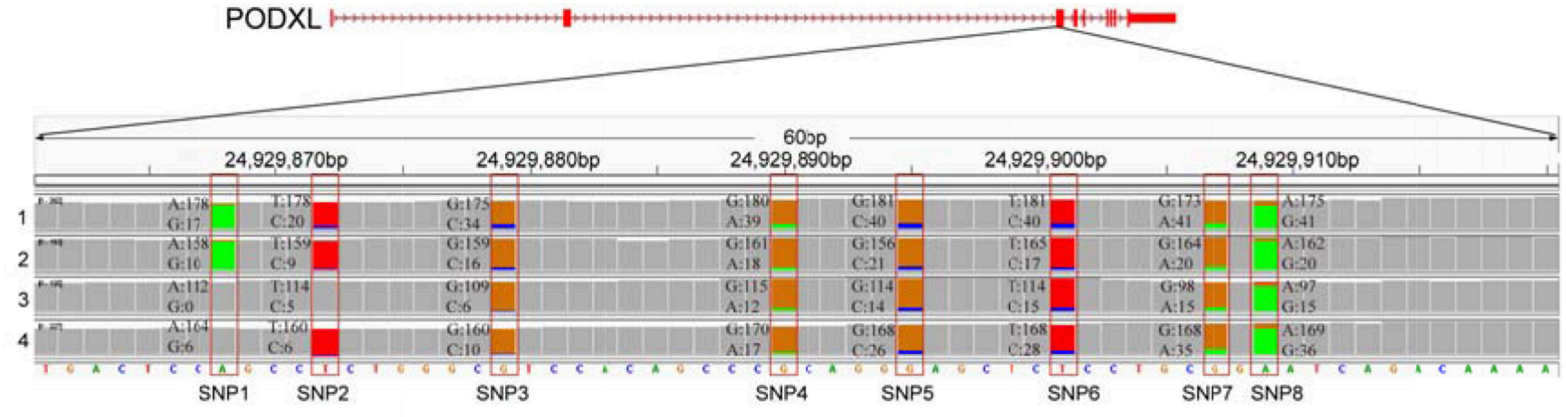
The allelic expression of eight SNPs containing ASE sites within the third exon of PODXL gene. The allele count information for each base was shown for the muscle tissues from four individuals (tracks 1–4). The colored bar indicates ASE sites with different colors representing the counts of corresponding alleles.

We further examined the ASE genes under selection. Thirty-eight genes with ASE from the six tissues were identified as being under directional selection, implying that they are involved in biologically essential functions, and these genes were therefore defined as core-ASE genes. The largest number of core-ASE genes (13) was found in the liver, where four genes were associated with metabolism (*LOC102191011, LOC102177333, LOC102173339*, and *LOC102191297*), and another four were associated with the immune system (*TAP1, LOC102189753, LOC102171351*, and *TDO2*). In skin, there were four core-ASE genes, including *TNXB*, which is related to the pathogenesis of systemic lupus erythematosus and *RPS8*, which is involved in resistance to hemoprotozoal diseases that cause economic losses among sheep and goats in tropical and subtropical regions. A small number of core-ASE genes were also found in bone (6) and the mammary glands (3), as shown in (Figure 3). Notably, two core-ASE genes (*PODXL* and *RPS8*) were present in at least two tissues, and in the *PODXL* gene, the ASE phenomenon was detected in eight SNPs as soon as the SNPs became heterozygotic in individuals, except for SNP1 and SNP2 in sample 1 (Figure 4).

## Discussion

Studies in recent years have shown that ASE analysis is an efficient tool for identifying causative genetic variations. However, few studies have conducted ASE analysis in livestock, partially due to the high costs of generating hybrids. In this study, we generated F1 hybrids of two diverse breeds and then explored ASE genes in these hybrids. We further combined genomic selection signals and ASE analysis to gain insight into the genomic contributions underlying phenotypic differences and local adaptability to different environments. It should be noted that although we did not have reciprocal cross for the identification of imprinted genes, we excluded the candidates based on previously identified imprinted genes as much as possible in other species (human, mice, cattle and sheep).

Genetic diversity patterns and overall low heterozygosity are commonly used statistical methods for detecting genomic regions related to selection in domesticated animals. To detect putative selective loci in the present study, we performed sequencing in six Chinese Yunling black goats (representative of Chinese southern domestic black goats) and four Nubian goats and calculated the corresponding Fst and Hp values. Top 1% of the selection signals does not automatically mean positive selection, but they could narrow down our list of candidate genes. Thus, we identified a total of 521 genes showing population differentiation that potentially contribute to the phenotypic and adaptation traits of the goats. However, the genetic differentiation between the two breeds may be due to breeding, evolutionary and management history. Furthermore, the adaptation and phenotypic differences of the goats may be mediated by a complex network of genes that act in tandem, rather than by the action of single candidate gene (Lv et al., 2014; Kim et al., 2015). It is therefore difficult to directly draw conclusions regarding the genetic mechanisms underlying the observed traits based only on genomic selection signals. With only 3 F1, 6 Yunling and 4 Nubian WGS, it looks sample size (*n* = 13) was too small to obtain reliable estimates. But our objective is to understand the phenotypic difference among Yunling and Nubian breed by tracing and quantifying allele-specific expression (ASE) in F1 hybrid individuals. We are more concerned about the ASE results since this part is based on RNA-seq data from multiple tissues. The selection signal analysis indeed could have a large number of false positive results. However, our reported selection signals were only used to narrow down the candidate genes from ASE analysis by choosing the overlapping genes. Therefore, although the selection signals would contain large proportion of false positives, it would not affect our interpretation of the main results.

ASE is an important source of phenotypic diversity. The phenotypic traits of F1 hybrids are determined by the coordinated expression of alleles from both parents. In F1 hybrids, the two alleles from the parents will be exposed to the same trans-acting factors; thus, allelic-specific expression can be attributed only to differences in the cis-acting factors. When the parents show high genetic divergence, we can easily distinguish the origin of the two alleles based on their inherited SNPs from their parents. In the characterization of ASE in F1 hybrids, both alleles within the same cellular environment are evaluated, directly revealing cis-acting genetic variation in transcript accumulation (Springer and Stupar, 2007; Perumbakkam et al., 2013; Aguilar-Rangel et al., 2017). To our knowledge, this is the first ASE analysis of an F1 hybrid generated from a cross between two different breeds of goats.

We set up an experimental cross designed to characterize the divergence of gene regulation between Chinese Yunling black and Nubian goats via RNA-Seq analysis in six tissues of nine F1 hybrids, to identify candidate genes underlying local adaptation. Hundreds of ASE genes were found in different tissues, and a small proportion of these genes (core-ASE) were further shown to experience directional selection. These core-ASE genes are related to many essential biological processes, including metabolism (*LOC102191011, LOC102177333, LOC102173339*, and *LOC102191297*) (Elens et al., 2012; Bertaggia et al., 2017; Buermans Henk et al., 2017), immune responses (*TAP1, LOC102189753, LOC102171351*, and *TDO2*) (Grassmann et al., 2016; Hanalioglu et al., 2017; Kota et al., 2017), and the adaptation to hot and humid environments (RPS8 and TNXB) (Wei and Hemmings, 2003). The identification of these genes will help to explain the phenotypic differences and genetic mechanisms underlying the adaptation of the two representative goat breeds examined in this study and will supply a theoretical basis for crossbreeding and the improvement, breeding and the selection of local goats.

## Data Availability

The whole genome sequence data and RNA-seq data have been deposited in NCBI short read archive under study PRJNA504493 and PRJNA485657, respectively.

## Ethics Statement

All animal experiments were approved by the Institutional Animal Care and Use Committee at the College of Animal Science and Technology, Northwest A & F University. All experimental goats were housed in Black Goat Farm of Guangxi Institute of Animal Science, Nanning, China.

## Author Contributions

YJ, YC, and JL designed the experiment. YC fed the goats and collected the experimental tissues. HX and RL contributed to analyzing the data and interpreting the results. YC wrote the manuscript with input from all the authors. SG and NC participated in designing the structure of the article. All the authors read and approved the final manuscript.

### Conflict of Interest Statement

The authors declare that the research was conducted in the absence of any commercial or financial relationships that could be construed as a potential conflict of interest.
