# Supplementary material for: Genetic Basis of Phenotypic Differences Between Chinese Yunling Black Goats and Nubian Goats Revealed by Allele-Specific Expression in Their F1 Hybrids
Source: Front Genet. 2019 Mar 5;10:145. doi: 10.3389/fgene.2019.00145 (PMC6411798; doi:10.3389/fgene.2019.00145)
Supplement: Supplementary file 1 [file Presentation_1.pdf]

Supplementary TABLE 1 | Genes with ASE that overlap with known imprinted genes

Gene	Species				Expressed allele
	Human	Mouse	Sheep	Cattle	
IGF2R	√	√	√	√	Maternal
GRB10	√	√	√		Maternal
ALDH1L1	√				Maternal
OBSCN	√				Paternal

Supplementary TABLE 2 | Comparison of ASE Gene and Normal Gene Expression

	ASE-average fpkm	ASE-average number	Normal-average fpkm	Normal-average number
**Liver**	**576.03**	144	**19.49**	15785
**Bone**	**146.15**	107	**19.66**	19908
**Muscle**	**645.53**	78	**20.99**	19187
**Fat**	**45.22**	97	**20.9**	19670
**Mammary**	**70.88**	124	**19.88**	20304
**Skin**	**162.66**	127	**19.22**	20347

Supplementary TABLE 3 | Genes with ASE that have an overlapping situation in six tissues

Tissue number	Tissue	Gene number	Gene
6	bone, fat, liver, mammary, muscle, skin	5	*HLA-A, HLA-B, HLA-DQA1, HLA-DQB1, LOC106503915*
5	bone, fat, liver, muscle, skin	1	*SH3TC1*
	bone, fat, mammary, muscle, skin	5	*SGCB, SPCS3, TUBA1A, PODXL, LOC108633505*
4	bone, fat, liver, mammary	1	*LOC102172184*
	bone, fat, liver, skin	1	*HEBP1*
	fat, liver, mammary, muscle	1	*ENPP5*
	fat, liver, mammary, skin	1	*LOC102185917*
	bone, mammary, muscle, skin	1	*RPS8*
	bone, fat, mammary, skin	1	*LOC102185917*
	fat, mammary, muscle, skin	2	*TMEM43, AHNAK*
3	bone, fat, liver	2	*C3, TAP1*
	bone, liver, mammary	1	*XPC*
	liver, mammary, muscle	1	*GHR*
	liver, muscle, skin	1	*LOC102173702*
	fat, liver, mammary	2	*LOC102172205, LOC102176375*

	bone, fat, mammary	2	*LOC102189675, TNFAIP2*
	fat, mammary, muscle	1	*LOC102178315*
	mammary, muscle, skin	1	*LC102169149*
	fat, mammary, skin	3	*CLN5, LOC102169730, SEPN1*
2	bone, liver	3	*CD5L, LOC102169730, LOC108637533*
	liver, muscle	4	*EGFR, DEPTOR, MRPL17, LOC108637533*
	fat, liver	1	*C4A*
	liver, mammary	6	*HSPA13, LOC102169084, LOC102181292, LOC102174841, LOC102169190, LOC108638035*
	liver, skin	1	*COL18A1*
	bone, muscle	1	*GMPR*
	bone, fat	5	*LOC106501822, RGCC, LOC102174023, LOC108633303, LOC108638310*
	bone, mammary	4	*STK19, COL6A1, LOC102184629, LOC106503943*
	bone, skin	1	*SLC43A1*
	fat, muscle	2	*AMOTL1, FAM127A*
	fat, mammary	13	*LOC102180881, TGM2, LOC108633235, LOC102175469, CD36, KIAA1456, AGGF1, DDX58, LOC108634436, LOC102175225, LOC102176116, LOC102184922, LOC102172138*

	fat, skin	4	*LOC102186320, FARP2, SLC40A1, NPC1*
	mammary, skin	8	*CPNE8, TUBB2A, LYRM9, LOC102172005, METTL22, PAPLN, LOC102176726, LOC108636992*
1	liver	112	
	bone	73	
	muscle	53	
	fat	44	
	mammary	65	
	skin	91	

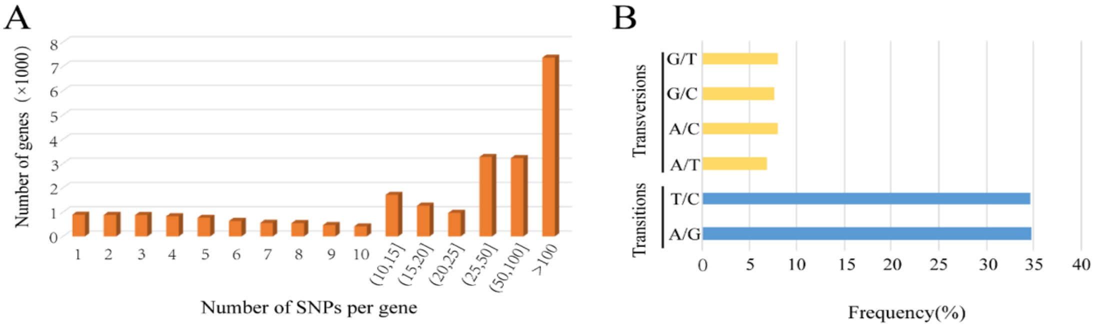

Supplementary Figure 1. SNP identification among Nubian goats, Chinese Yunling black goats and their hybrid F1 offspring. (A) SNP distribution according to the number of SNPs per gene. (B) Frequency of different substitution types in the identified SNPs.
