# Supplementary material for: Genetic Basis of Phenotypic Differences Between Chinese Yunling Black Goats and Nubian Goats Revealed by Allele-Specific Expression in Their F1 Hybrids
Source: Front Genet. 2019 Mar 5;10:145. doi: 10.3389/fgene.2019.00145 (PMC6411798; doi:10.3389/fgene.2019.00145)
Supplement: Supplementary file 2 [file Data_Sheet_1.pdf]

```python
# -*- coding: utf-8 -*-
#!/usr/bin/env python3

import sys
import os
import argparse
import time
import re

def GetCommandLine():
	CommandLine='python3 {0}'.format(' '.join(sys.argv))
	return(CommandLine)
LogFile=None
def log(LogInfo):
	'''
	Output the LogInfo to log file
	'''
	global LogFile
	if sys.platform == 'linux':
		CurrentFolder=os.getcwd()
		LogFileName=re.split('/|\\\\',sys.argv[0].strip())
		LogFileName=LogFileName[-1].split('.')
		LogFileName='{0}/{1}.log'.format(CurrentFolder,LogFileName[0])
		if LogFile:LogFile.write(LogInfo+'\n')
		else:
			LogFile=open(LogFileName,'w')
			LogFile.write(LogInfo+'\n')
	else:
		print(LogInfo)
def LoadSnp():
	'''
	1	612	T	C
	1	638	A	C
	1	681	G	C
	'''
	SnpDict = {}
	ChrDict = {}
	SnpList = []
	for line in args.SnpList:
		if line.startswith('#'): continue
		line = line.strip().split()
		index = '{0}-{1}'.format(line[0],line[1])
		SnpDict[index] = [line[2],line[3]]
		ChrDict[line[0]] = None
```

```
            SnpList.append(index)
    return SnpList,SnpDict,ChrDict
def RelocateReadsPos(pos,pattern,read,quality):
    '''
    49M             TTATCCATTGACACTTTACCATTCCCAACCCCAAGCTCTTCAACATCAA
    13M5D36M        TTATCCATTGACA    -----    CTTTACCATTCCCAACCCCAAGCTCTTCAACATCAA
    20M2I27M        TAGCTCCTATTAGAGCAAAT    (AA)    AAGAGATGGAAATAGTTTGTTTTTCAG
    3S27M19S        (ATT)    AGTAAATAGTACATCATTGGCACTCTT    (TATTGGATTGCAAAATAAA)
    7H49M7H         TTATCCATTGACACTTTACCATTCCCAACCCCAAGCTCTTCAACATCAA
    '''
    PosList=[]
    PatternNum=re.findall('\d+',pattern)
    PatternStr=re.findall('\D+',pattern)
    for i in range(len(PatternNum)):
        if PatternStr[i]=='S':
            read=read[int(PatternNum[i]):]
            quality=quality[int(PatternNum[i]):]
        elif PatternStr[i]=='M':
            PosList.append([pos,read[0:int(PatternNum[i])],quality[0:int(PatternNum[i])]])
            pos=pos+int(PatternNum[i])
            read=read[int(PatternNum[i]):]
            quality=quality[int(PatternNum[i]):]
        elif PatternStr[i]=='N':
            pos=pos+int(PatternNum[i])
        elif PatternStr[i]=='D':
            pos=pos+int(PatternNum[i])
        elif PatternStr[i]=='I':
            read=read[int(PatternNum[i]):]
            quality=quality[int(PatternNum[i]):]
        elif PatternStr[i]=='H':
            pass
    return PosList
def GetPosState(pos,PosInfo,SnpDict,RefDict,AltDict,OtherDict):
    if PosInfo == SnpDict[pos][0]:
        RefDict[pos]=RefDict.get(pos,0)+1
    elif PosInfo == SnpDict[pos][1]:
        AltDict[pos]=AltDict.get(pos,0)+1
    else:
        OtherDict[pos]=OtherDict.get(pos,0)+1
def GetSnpCountFromBam():
    '''
    M_ST-E00522:37:HFH23ALXX:3:2114:1905:28136    16    1    415    0
36M    *    0    0    AACTACTGTACATTTGCACTCATTTCACATGCCAGT
    M_ST-E00522:37:HFH23ALXX:3:1120:4990:39001    0    1    3433    25
```

```
42M	*	0	0	GTGTGGTGTGGTGTGGTGTGTGTGGTGTAATATGTGTGGTGT
	M_ST-E00522:37:HFH23ALXX:3:2220:29031:4139	16	1	4407	0
60M	*	0	0
GTGTGTGTAGGGGTGTGTGTGGTGTGATGTGTGTGTAGGGGTG
	'''
	RefDict = {}
	AltDict = {}
	OtherDict = {}
	SnpList,SnpDict,ChrDict = LoadSnp()
	BamFile = os.popen('samtools view {0}'.format(args.bam))
	for reads in BamFile:
		reads = reads.strip().split()
		if not reads[2] in ChrDict: continue
		if reads[5] == '*': continue
		if int(reads[4]) < args.rq: continue
		ReadsPos=RelocateReadsPos(int(reads[3]),reads[5],reads[9],reads[10])
		for i in range(len(ReadsPos)):
			for n in range(0,len(ReadsPos[i][1])):
				ChrPos = '{0}-{1}'.format(reads[2],ReadsPos[i][0]+n)
				if not ChrPos in SnpDict: continue
#				  print(ord(ReadsPos[i][2][n])-33)
				if ord(ReadsPos[i][2][n])-33 < args.pq: continue
				PosInfo=ReadsPos[i][1][n]
				GetPosState(ChrPos,PosInfo,SnpDict,RefDict,AltDict,OtherDict)
	for SnpPos in SnpList:

args.output.write('{0}\t{1}\t{2}\t{3}\n'.format(SnpPos.replace('-','\t'),RefDict.get(SnpPos,0),AltDic
t.get(SnpPos,0),OtherDict.get(SnpPos,0)))
def main():
	print('Running...')
	log('The start time: {0}'.format(time.ctime()))
	log('The command line is:\n{0}'.format(GetCommandLine()))
	GetSnpCountFromBam()
	log('The end time: {0}'.format(time.ctime()))
	print('Done!')
##############################Argument
parser=argparse.ArgumentParser(description=print(__doc__),formatter_class=argparse.Argumen
tDefaultsHelpFormatter)
parser.add_argument('-s','--SnpList',metavar='File',dest='SnpList',help='Snp	List
file',type=open,required=True)
parser.add_argument('-b','--bam',metavar='File',dest='bam',help='Bam
file',type=str,required=True)
parser.add_argument('-q','--MinReadsQuality',dest='rq',help='Min	Reads
Quality',type=int,default=30)
```

```
parser.add_argument('-Q','--MinPosQuality',dest='pq',help='Min                                    Pos
Quality[0-93]',type=int,default=30)
parser.add_argument('-o','--Output',metavar='File',dest='output',help='Output
file',type=argparse.FileType('w'),required=True)
args=parser.parse_args()
############################
if __name__=='__main__':
	main()
```
